# Vaginal microbes alter epithelial transcriptomic and epigenomic modifications providing insight into the molecular mechanisms for susceptibility to adverse reproductive outcomes

**DOI:** 10.21203/rs.3.rs-3580132/v1

**Published:** 2023-11-16

**Authors:** Michal Elovitz, Lauren Anton, Ana Cristancho, Briana Ferguson, Andrea Joseph, Jacques Ravel

**Affiliations:** Icahn School of Medicine at Mount Sinai; Perelman School of Medicine at the University of Pennsylvania; Children’s Hospital of Philadelphia, Perelman School of Medicine at the University of Pennsylvania; University of Pennsylvania; Ichan School of Medicine at Mount Sinai; University of Maryland, Baltimore

**Keywords:** cervix, epithelial cells, lactobacillus crispatus, gardnerella vaginalis, RNA-seq, anti-microbial peptides, NLRP3 inflammasome, ATAC-seq, chromatin, women’s health

## Abstract

The cervicovaginal microbiome is highly associated with women’s health with microbial communities dominated by *Lactobacillus* spp. being considered optimal. Conversely, a lack of lactobacilli and a high abundance of strict and facultative anaerobes including *Gardnerella vaginalis*, have been associated with adverse reproductive outcomes. However, the molecular pathways modulated by microbe interactions with the cervicovaginal epithelia remain unclear. Using RNA-sequencing, we characterize the *in vitro* cervicovaginal epithelial transcriptional response to different vaginal bacteria and their culture supernatants. We showed that *G. vaginalis* upregulated genes were associated with an activated innate immune response including anti-microbial peptides and inflammasome pathways, represented by NLRP3-mediated increases in caspase-1, IL-1β and cell death. Cervicovaginal epithelial cells exposed to *L. crispatus* showed limited transcriptomic changes, while exposure to *L. crispatus* culture supernatants resulted in a shift in the epigenomic landscape of cervical epithelial cells. ATAC-sequencing confirmed epigenetic changes with reduced chromatin accessibility. This study reveals new insight into host-microbe interactions in the lower reproductive tract and suggest potential therapeutic strategies leveraging the vaginal microbiome to improve reproductive health.

## Introduction

The female lower reproductive tract is comprised of a complex ecosystem made up of host epithelial and immune cells, microorganisms including the microbiome (bacteria) ^1^, mycobiome (fungi) ^2^, virome (viruses) ^3,4^ and their metabolites. The cervicovaginal microbiome has become the focus of many studies due to its highly integrated and complex role in reproductive health and disease. Using high throughput 16S rRNA gene sequencing, the vaginal microbiota composition has been well characterized in both non-pregnant and pregnant individuals ^5–7^. The vaginal microbiome has traditionally been defined by the presence or absence of *Lactobacillus* spp. ^5,8,9^. Cervicovaginal microbial communities dominated by *Lactobacillus* spp. are generally considered optimal and associated with positive reproductive health outcomes, while those lacking lactobacilli and comprising a wide array of strict and facultative anaerobes are associated with a diverse spectrum of adverse gynecological and reproductive outcomes including infertility ^10,11^, sexually transmitted infections (STIs) (human papilloma virus (HPV) ^12^ and human immunodeficiency virus (HIV)) ^13^, and pregnancy complications (preterm birth) ^14,15^. Although the cervicovaginal microbiome is less taxonomically diverse than those found at other body sites, the identification of con-specific genotypes cohabitating in the vaginal microbiome adds to the complexity of these communities ^16^. Despite these advances in vaginal microbiome characterization and its association with clinical outcomes, there is a paucity of data regarding the precise mechanisms by which vaginal microbes modulate the functioning of host epithelia and promote adverse health outcomes.

The vaginal microbiome interacts with the epithelial barriers of the cervicovaginal space. The cervicovaginal epithelial barrier is unique as the epithelial cells that line this space have different embryological origins resulting in distinct cell-specific functions including mucus production ^17–20^. The cervicovaginal epithelial barrier acts as the primary site of entry for invading pathogens and disruption of the barrier is associated with adverse health outcomes including increased risk of STI (*e.g*., chlamydia, gonorrhea. HIV) acquisition ^21,22^. Undoubtedly, revealing the molecular mechanisms by which vaginal microbes modulate host responses in the lower reproductive tract is necessary to understand how vaginal microbial communities drive reproductive health and disease. Therefore, the objectives of this study were to 1) perform discovery-based RNA-sequencing to identify genes and functional pathways in cervical and vaginal epithelial cells that are altered after exposure to *G. vaginalis* or *L. crispatus* and their culture supernatants, 2) elucidate and speciate the immune pathways activated by *G. vaginalis* and 3) identify molecular mechanisms by which *L. crispatus* optimizes cervical and vaginal epithelial function.

## Results

### Cervicovaginal epithelial cell gene transcription and associated functional pathways are differentially modulated by L. crispatus, G. vaginalis and their culture supernatants

**Figure 1:**
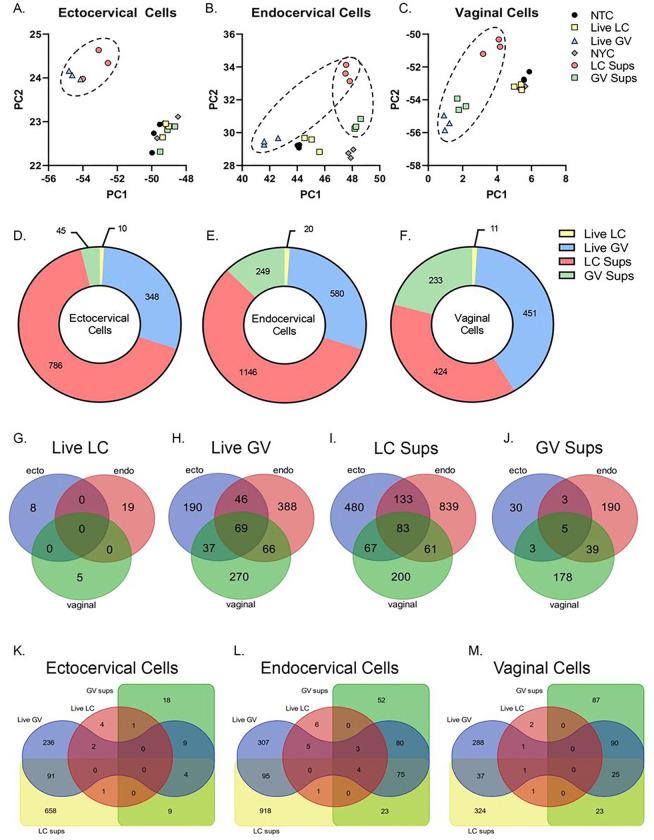
RNA-seq identified differentially expressed genes in cervicovaginal epithelial cells after 24 hr exposure to live bacteria or culture supernatants from *L. crispatus* or *G. vaginalis*. Principle component analysis (PCA) plots showing gene expression modulation in ectocervical (A), endocervical (B) and vaginal (C) cells exposed to either live *G. vaginalis* (vs NTC control) or *L. crispatus* culture supernatants (vs NYCIII control). The total number of differentially expressed genes (adj. p<0.05, Log2FoldChange ≥ 1 and ≤ −1) in each exposure group by cell line (D-F). The number of overlapping differentially expressed genes between cervicovaginal cell types for each bacterial exposure (G-J). The number of overlapping differentially expressed genes between bacterial exposures within each cervicovaginal cell types (K-M).

Exposure of cervical and vaginal epithelial cells to live *L. crispatus* or *G. vaginalis* or their bacteria-free culture supernatants resulted in significant differences in gene expression profiles. In ectocervical, endocervical and vaginal epithelial cells, PCA plots show distinctive clustering by bacterial exposure and indicates the greatest differences (Fig. 1A–C) between exposures. Exposure to *L. crispatus* culture supernatants and live *G. vaginalis* were noted to be most distant from their respective controls (NYCIII for culture supernatants and NTC for live bacteria) (Fig. 1A–C). Gene expression profiles appeared to be epithelial cell specific as exposure to *G. vaginalis* culture supernatant resulted in clustering that was different from the NYCIII control in endocervical and vaginal cells that was not seen in ectocervical cells (Fig. 1A–C). To avoid false discovery, we then identified differentially expressed genes with an adjusted p-value < 0.05 and a Log2 fold change of greater than 1 or less than − 1 (Supplemental Table 1). Using these criteria, in all three cell types, the number of differentially expressed genes is highest after exposure to *L. crispatus* culture supernatants followed by live *G. vaginalis* and *G. vaginalis* supernatants (Fig. 1D–F). In contrast, very few genes were differentially expressed in all three epithelial cell types after exposure to live *L. crispatus* (Fig. 1G). Figure 1G–J depicts the number of overlapping differentially expressed genes between cervicovaginal cell types for each bacterial exposure. For *G. vaginalis* exposures (live or supernatant), endocervical and vaginal epithelial cells have the highest number of overlapping differentially expressed genes, while exposure to *L. crispatus* culture supernatants illicit the most overlapping differentially expressed genes in ectocervical and endocervical epithelial cells (Supplemental Table 2). Additional comparisons were performed within each cervicovaginal epithelial cell type to identify commonly modulated genes by these different bacterial exposures (Fig. 1K–M, Supplemental Table 3). From these analyses, we were able to identify unique genes where expression was altered by both live *G. vaginalis* and *L. crispatus* culture supernatant but were differentially regulated; thus, demonstrating specific molecular effects of these bacterial exposures on regulating cervicovaginal epithelial cell transcription (Supplemental Table 4, 5, 6). In summary, genes that were upregulated by exposure to *G. vaginalis* and downregulated by *L. crispatus* point towards a pro-inflammatory response. On the other hand, those upregulated by exposure to *L. crispatus* and downregulated by *G. vaginalis* seem to be involved in transcription, epigenetic modifications, cell-cell adherence, and anti-inflammatory functions.

**Figure 2:**
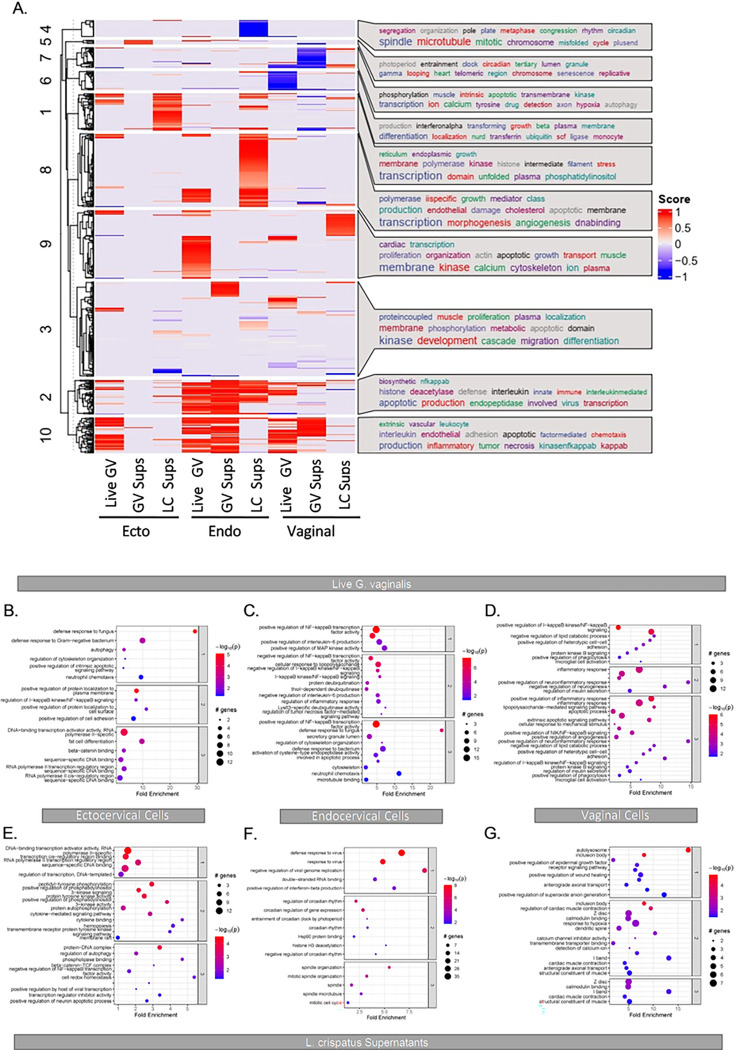
Differential clustering of significant differentially expressed genes (adj. p<0.05, Log2FoldChange ≥ 1 and ≤ −1) between exposure groups and across cervicovaginal cell lines reveal modulation of functional pathways (A). Functional pathway analysis (B-G) of RNA-seq data for ectocervical (B, E), endocervical (C, F) and vaginal (D, G) epithelial cells exposed to live *G. vaginalis* or *L. crispatus* culture supernatants.

Functional pathway analysis using Gene Ontology terms identified specific pathways of interest between bacterial exposures. Live *G. vaginalis* and *G. vaginalis* culture supernatant upregulated genes were mostly associated with inflammation functional pathways (Fig. 2A, 2B–D). Exposure to *L. crispatus* culture supernatant was associated with upregulation of transcriptional functional pathways including histone modifications, RNA polymerase II transcription and DNA binding (Fig. 2A, 2E–G). Live *L. crispatus* modulated the expression of too few genes to perform functional pathway analysis. Since 1) live *G. vaginalis* and its culture supernatant modulated the same functional pathways and 2) *L. crispatus* culture supernatant resulted in the highest number of differentially expressed genes while live *L. crispatus* had a minimal effect on epithelial cell gene expression, we chose to focus on exposures of live *G. vaginalis* and *L. crispatus* culture supernatant for further analysis.

### Live G. vaginalis and its culture supernatant differentially up-regulate anti-microbial peptide gene expression and increase protein levels

**Figure 3:**
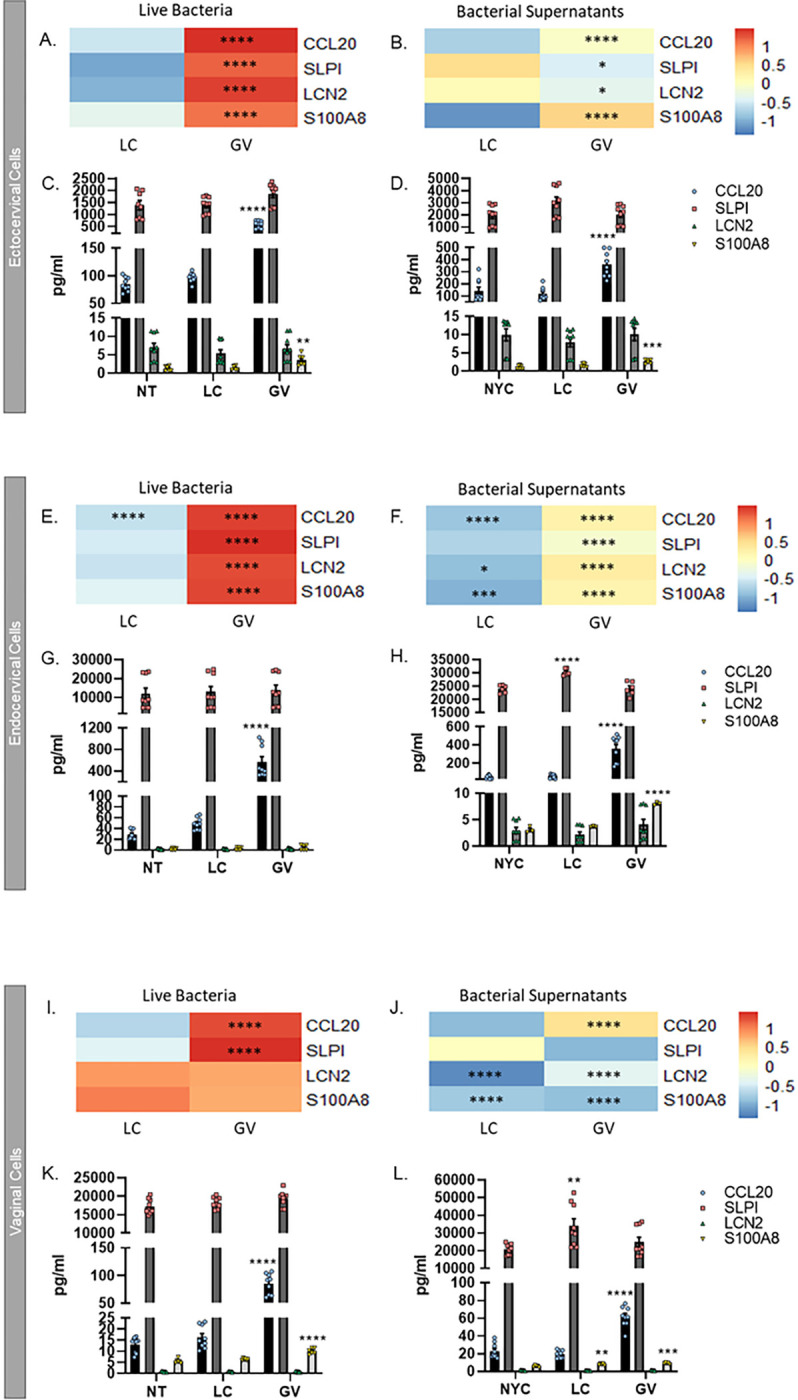
Anti-microbial peptide gene expression and proteins are significantly increased after cervicovaginal cell exposure to live bacteria or culture supernatants from *G. vaginalis*. RNA-seq identified CCL20, SLPI, LCN2 and S100A8 as being significantly upregulated by live *G. vaginalis* and its supernatants in ectocervical (A, B), endocervical (E, F) and vaginal (I, J) epithelial cells. ELISAs further identified that these anti-microbial peptides were increased after live *G. vaginalis* (C, G, K) or *G. vaginalis* culture supernatant exposure (D, H, J). Values are mean ± SEM. Asterisks over the individual bars represent comparisons to control; asterisks over solid lines represent comparisons between treatment groups. *p<0.05, **p<0.01, ***p<0.001, ****p<0.0001.

RNA-seq functional pathway analysis identified “defense response to a bacterium” along with various inflammation-based pathways as Gene Ontology terms of significance after live *G. vaginalis* exposure (Fig. 2B–D). The specific genes associated with these pathways included the anti-microbial peptides, Chemokine Ligand 20 (CCL20), Secretory Leukocyte Peptidase Inhibitor (SLPI), Lipocalin 2 (LCN2) and S100 Calcium Binding Protein 8 (S100A8/A9, Calgranulin). Our data show that all four of these genes were significantly upregulated (adjusted p < 0.05) by live *G. vaginalis* exposure in ectocervical and endocervical cells with only CCL20 and SLPI being upregulated in vaginal cells (Fig. 3A, 3E, 3I). After exposure to *G. vaginalis* culture supernatants, CCL20 was the only transcript upregulated in all three cell lines (adjusted p < 0.05, Fig. 3B, 3F, 3J). In ectocervical cells, in addition to CCL20, S100A8 gene expression was upregulated after *G. vaginalis* culture supernatant exposure (adjusted p < 0.05, Fig. 3B). In endocervical cells, all four anti-microbial peptide genes were significantly upregulated by *G. vaginalis* culture supernatant (adjusted p < 0.05, Fig. 3F). Only expression of CCL20 was upregulated in vaginal cells after *G. vaginalis* culture supernatant exposure (adjusted p < 0.05, Fig. 3J). ELISAs for these antimicrobial peptides confirmed overexpression of CLL20 and S100A8 (both p < 0.05), but not SLPI and LCN2, which remained unchanged, after exposure to *G. vaginalis* (Fig. 3C, 3G, 3K) and *G. vaginalis* culture supernatants (Fig. 3D, 3H, 3L).

### Down-regulation of anti-microbial peptide gene expression, but not protein, by live L. crispatus and its culture supernatant

Live *L. crispatus* did not affect the gene expression of the four anti-microbial peptides measured (Fig. 3A, 3E, 3I), except that CCL20 mRNA was downregulated (p < 0.0001) in endocervical cells; however, the amount of CCL20 protein was not altered at this same time point (Fig. 3G). *L. crispatus* culture supernatants downregulated the gene expression of LCN2 (p < 0.05) and S100A8 (p < 0.001) in endocervical and vaginal cells (Fig. 3F and 3J), but not ectocervical cells (Fig. 3B). Additionally, CCL20 (p < 0.0001) gene expression was downregulated in endocervical cells (Fig. 3F). However, this decrease in anti-microbial gene mRNA expression in endocervical and vaginal cells was not associated with a change in their associated proteins (Fig. 3H and 3L).

### G. vaginalis activates an inflammasome-mediated immune response in cervicovaginal epithelial cells

As noted prior, cervicovaginal cells exposed to *G. vaginalis* resulted in the differential expression of genes significantly associated with inflammation-related functional pathways (Fig. 2A, 2B–2D). These genes, included those coding for multiple chemokines and cytokines (*e.g*., IL-8, IL-6, IL-1α and TNF), have been investigated previously ^23^. Novel to this work, we identified several inflammasome-mediated genes that were significantly upregulated after *G. vaginalis* exposure including NLRP3, NLRP1, IL-1β and caspase-1 (adjusted p < 0.05) (Table 1). The gene expression changes for all detectable inflammasome-associated genes are listed in Supplemental Table 7. To determine if inflammasome activation contributes to the inflammatory response seen after *G. vaginalis* exposure in cervicovaginal epithelial cells, we performed a bacterial dose response experiment (1×10^5^–1×10^7^ CFUs/ml) for both *L. crispatus* and *G. vaginalis*. Caspase-1, IL-1β, and lactate dehydrogenase (LDH, a marker of cell death) release were significantly increased (p < 0.0001) in a dose-dependent manner from ectocervical, endocervical and vaginal cells after exposure to *G. vaginalis* but not *L. crispatus* (Supplemental Fig. 1).

### G. vaginalis activates the NLRP3 inflammasome in THP monocytes

To determine the specificity of NLRP3 inflammasome activation in mediating *G. vaginalis*-induced increases in IL-1β, caspase-1 or cell death in cervicovaginal epithelial cells, we treated monocytes, THP1-Null2 cells or THP1-NLRP3-KO (knock out) cells, with LPS (positive control), live *L. crispatus* (control for live *G. vaginalis*) or live *G. vaginalis*. Exposure to live *G. vaginalis*, but not L. crispatus, resulted in a significant increase in caspase-1 (p < 0.001, Fig. 6A) and IL-1β (p < 0.0001, Fig. 6B) in THP1-Null2 cells but not in THP-NLRP3-KO cells (p < 0.001). Cell death (LDH, Fig. 6C) remained unchanged after bacterial exposure in both THP1 cell types. Additionally, we treated THP1-Null2 cells with glybenclamide (glyburide) ^24^, a specific inhibitor of NLRP3 (with no effects on NLRC4 or NLRP1). Glybenclamide treatment significantly reduced caspase-1 (p < 0.05) and IL-1β (p < 0.0001, Fig. 6D and 6E). As with THP1-NLRP3-KO, no reduction in *G. vaginalis*-mediated cell death (LDH, Fig. 6E) was observed in THP1-Null2 cells treated with the NLRP3 inhibitor.

### G. vaginalis-mediated inflammasome activation in cervicovaginal epithelial cells is NLRP3 specific

Since *G. vaginalis* activates the NLRP3 inflammasome in THP1 monocytes, we determined if *G. vaginalis* could also activate an NLRP3-specific inflammasome in cervicovaginal epithelial cells. Glybenclamide treatment resulted in a significant reduction in *G. vaginalis*-mediated increases in caspase-1 (p < 0.01, Fig. 5A, 5D, 5G) and IL-1β (p < 0.05, Fig. 5B, 5E, 5H) in ectocervical, endocervical and vaginal cells, while *G. vaginalis*-induced cell death, as measured by LDH release, was unchanged (Fig. 5C, 5F, 5I).

### G. vaginalis activates the inflammasome-mediated immune response through caspase-1

**Figure 4:**
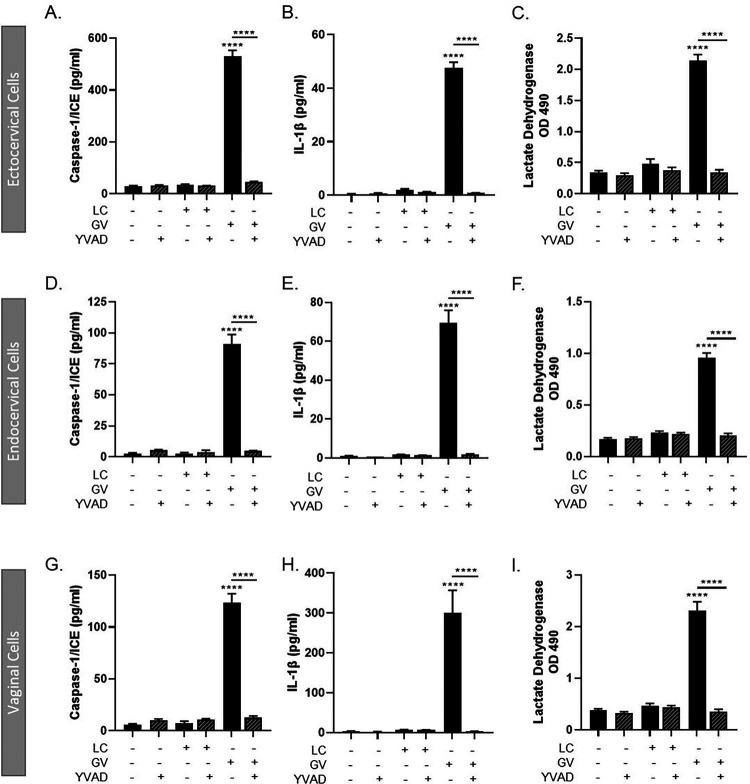
Live *G. vaginalis*, but not L. crispatus, exposure initiates canonical-inflammasome activation in ectocervical (A-C), endocervical (D-F) and vaginal (G-I) epithelial cells. Exposure to live *G. vaginalis* for 24hrs increases caspase-1 (A, D, G), IL-1β (B, E, H) and cell death as measured by lactate dehydrogenase (LDH) (C, F, I). The *G. vaginalis*-mediated increases in inflammasome-associated proteins are significantly reduced after pre-treatment with YVAD, a caspase-1 inhibitor. Values are mean ± SEM. Asterisks over the individual bars represent comparisons to non-treated control; asterisks over solid lines represent comparisons between treatment groups. ****p<0.0001

**Figure 5:**
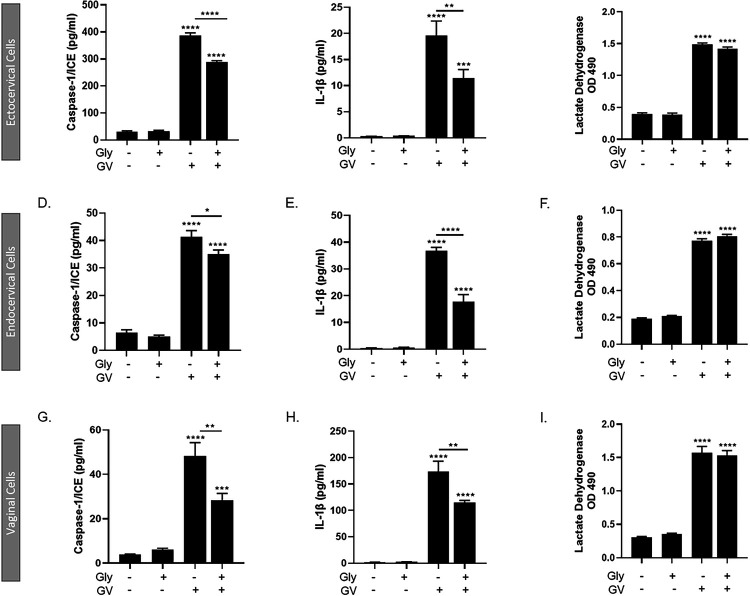
Inflammasome activation by *G. vaginalis* is specific to the NLRP3 inflammasome. Pre-treatment with glybenclamide, a specific NLRP3 inhibitor, was able to partially reduce the *G. vaginalis*-mediated increases in caspase-1 (A, D, G) and IL-1β (B, E, H) with no changes to cell death. Values are mean ± SEM. Asterisks over the individual bars represent comparisons to non-treated control; asterisks over solid lines represent comparisons between treatment groups. *p<0.05, **p<0.01, ***p<0.001, ****p<0.0001.

**Figure 6:**
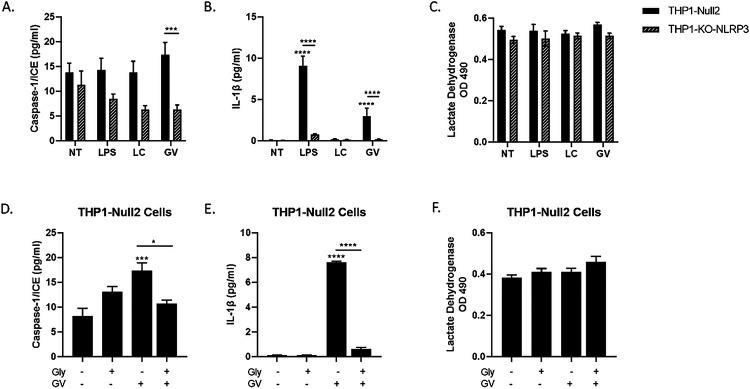
Live *G. vaginalis* induces inflammasome activation in THP-1 monocytes. Exposure to live *G. vaginalis* increases caspase-1 (A) and IL-1β (B) but not cell death (LDH) (C) in THP-null-2 cells. In THP-NLRP3 knockout cells the *G. vaginalis*-mediated increases in inflammasome-associated proteins are significantly reduced (A, B). LPS, included as a positive control, significantly increase IL-1β in THP-null-2 cells which was reduced in THP-NLRP3 knockout cells. Pre-treatment with glybenclamide, a specific NLRP3 inhibitor, significantly reduced the *G. vaginalis*-mediated increase in caspase-1 (D) and IL-1β (E). LDH was unchanged by glybenclamide (F). Values are mean ± SEM. Asterisks over the individual bars represent comparisons to control; asterisks over solid lines represent comparisons between treatment groups. *p<0.05, **p<0.01, ***p<0.001, ****p<0.0001.

Since inhibiting NLRP3 activation did not fully mitigate the *G. vaginalis*-mediated increases in IL-1β, caspase-1 or cell death in cervicovaginal epithelial cells, we sought to determine if inhibiting caspase-1 would fully block inflammasome activation. Using the irreversible caspase-1 inhibitor, Ac-YVAD-cmk ^25^, we determined that caspase-1 is an essential regulator of inflammasome activation by *G. vaginalis*. Inhibition of caspase-1 resulted in a significant reduction in the *G. vaginalis*-mediated increase in caspase-1 (p < 0.0001) (Fig. 4A, 4D, 4G), IL-1β (p < 0.0001) (Fig. 4B, 4E, 4H) and cell death (p < 0.0001) (LDH, Fig. 4C, 4F, 4I) in ectocervical, endocervical and vaginal cells after *G. vaginalis* but not *L. crispatus* exposure.

### L. crispatus culture supernatants alter chromatin accessibility

As most of the enriched pathways associated with differentially expressed genes in all three cervicovaginal cell types exposed to *L. crispatus* culture supernatants were related to alterations in histone and transcriptional regulation (Fig. 2A, 2E–G), we used assay for transposase-accessible chromatin high throughput sequencing (ATAC-seq) to ascertain whether these gene expression changes were associated with differences in chromatin accessibility ^26–28^. While all three cell types demonstrated substantial modulation of gene expression after exposure to *L. crispatus* culture supernatants, the prominence of upregulated epigenomic-associated functional pathways in ectocervical cells pointed to potential changes in chromatin accessibility in this cell type.

The number and percentage of aligned/unaligned reads were equal across all three cell types (Supplemental Fig. 2). Notably, the samples from the ectocervical cells had lower transcription start site (TSS) enrichment scores despite demonstrating similar quality control characteristics in alignment (Supplemental Fig. 3). There was a strong correlation between normalized read counts between conditions for each cell type (Supplemental Fig. 4).

**Figure 7:**
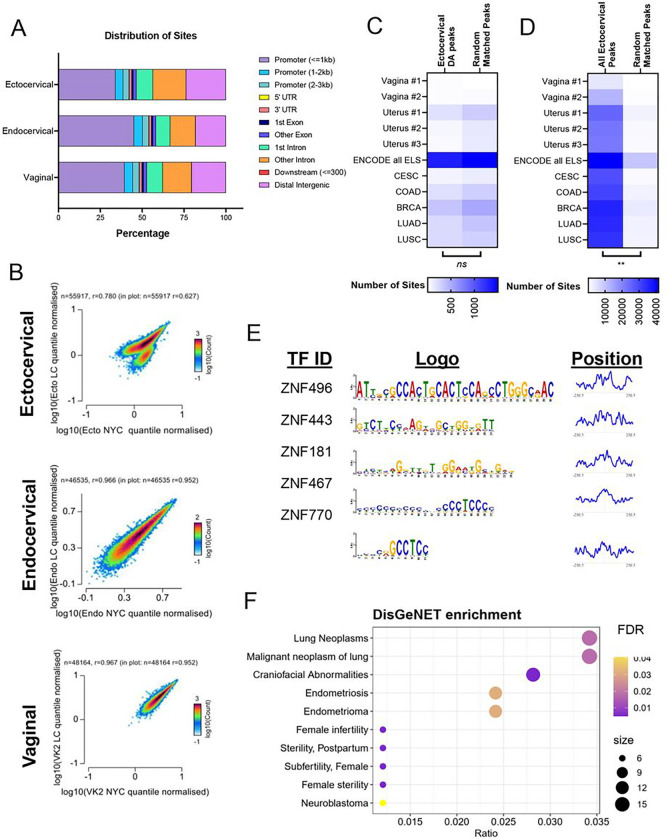
Chromatin accessibility was disrupted primarily in ectocervical cells exposed to *L. crispatus* supernatants. (A) Distribution of consensus sites by percentage. (B) Scatterplot of normalized counts between NYC and *L. crispatus* supernatant treatment by cell type-specific consensus peaks. (C) Distribution of differentially accessible sites in ectocervical cells across the genome. (D) Overlap of differentially accessible sites and matched number of random sites (E) all ectocervical peaks and matched number of random sites with published sites from ENCODE and multiple primary cancer specimens. (F) Motif analysis of downregulated differentially accessible sites with motif logo and graph of positional distribution based on the center of the peak of the top 5 motifs. (G) Bubble chart of DisGeNET enrichment of transcription factors identified by the motif analysis. DA: Differential Accessibility; ELS: Enhancer-like signatures; CESC: Cervical Squamous Cell Carcinoma; COAD: Colon Adenocarcinoma; BRCA: Breast Invasive Carcinoma; LUAD: Lung Adenocarcinoma; LUSC: Lung Squamous Cell Carcinoma.

We obtained a consensus peak set for each cell type (Ecto: 55,917 peaks, Endo: 46,535 peaks, VK2: 48,164 peaks). As expected, the majority of open chromatin peaks for all cell types were found in proximal promoter regions (< 1 kb) or intergenic regions (Fig. 7A). To determine if *L. crispatus* culture supernatant leads to cell-specific differences in chromatic organization, we compared the normalized read counts between different genomic regions. There was no difference in quantile normalized counts between the TSS and gene bodies for each cell type (Supplemental Fig. 5). However, only in the ectocervical cells, the consensus peak regions demonstrated nearly two discrete clusters (Fig. 7B). Endocervical and vaginal cells did not exhibit these two clusters of accessible sites (Fig. 7B).

We then tested whether there were regions of differential accessibility between the treatment conditions in the cell type-specific consensus peak sites. We detected 8,147 regions with differential accessibility profiles in *L. crispatus* supernatant-treated ectocervical cells, almost all showing reduced accessibility (8,125 with decreased accessibility, 22 with increased accessibility). There was only a fraction of sites differentially regulated in endocervical and vaginal cells (21 and 109 total sites, respectively). Notably, the distribution of differential accessibility sites was skewed towards an increase in the percentage of distal intergenic and intronic regions and a decrease in proximal promoter sites compared to the general distribution of all detected ectocervical peaks (Fig. 7C). We identified the genes neighboring the differentially regulated sites and sought to determine whether these genes overlapped with differentially expressed genes we had detected by RNA-sequencing after *L. crispatus* supernatant. Indeed, most genes with differential expression in ectocervical cells treated with *L. crispatus* supernatant had differential accessibility by ATAC-sequencing (683/762 genes)^29–31^.

Given the strong relationship between differentially expressed genes and differentially accessible chromatin, we sought to identify whether differentially accessible chromatin was more likely to be associated with previously identified putative tissue-specific regulatory regions or all identified human enhancer regions (enhancer-like sequences of different primary tissues described by the Encylopedia of DNA Elements (ENCODE) Project or open accessibility regions identified in different primary cancer specimens)^32–34^. Surprisingly, we discovered little overlap between differential accessibility in ectocervical cells and putative enhancer regions (Fig. 7D, Chi-squared test for trend *p* = 0.8334 in differentially accessible ectocervical peaks vs random matched control regions). However, ENCODE does not have specimens from primary cervical tissue so we could only compare the ectocervical signatures to specimens collected from the vagina and uterus. By contrast, there was a substantial overlap between all open chromatin regions identified in ectocervical cells and published likely enhancers (Fig. 7E, Chi-squared test for trend *p* = 0.0028 in all accessible ectocervical peaks vs random matched control regions).

Despite not being associated with known enhancer regions, motif analysis of the downregulated differentially accessible sites demonstrated enrichment for 497 transcription factor motifs with an FDR of 0.05 (Supplemental Table 8, Fig. 7F). The position of the top 5 identified motifs was at the center of the peak, consistent with the expected location of any true transcription factor binding^35^. Gene-disease enrichment analysis of these transcription factors demonstrated a marked enrichment of multiple pathways related to neoplasms, endometriosis and infertility, all pathologies potentially associated with *Lactobacillus*-dominated microbiota (Fig. 7G) ^36,37^. To determine if this enrichment was specific, we generated random lists of transcription factors from the same motif database and demonstrated only an overlapping enrichment in unrelated craniofacial anomalies, suggesting our findings are indeed specific (Supplemental Fig. 6).

## Discussion

Host-microbe interactions have been shown to govern health and disease in multiple biological systems. With this study, we now reveal unique molecular mechanisms involved in host-microbe interactions in the cervicovaginal space, thus addressing a large knowledge gap in reproductive health. These findings confirm the induction of diverse immune pathways in response to *G. vaginalis*, a facultative anaerobic bacteria associated with many gynecological disorders, including STI and HPV acquisition. Importantly, we found that *G. vaginalis* activates the NLRP3 inflammasome in cervicovaginal epithelial and immune cells. Further advancing our understanding of host-microbe interactions in the cervicovaginal space, we report that exposure to *L. crispatus* culture supernatants results in epigenetic modifications in ectocervical cells. Collectively, these studies demonstrate the complexity of host-microbe interactions with divergent effects between live bacteria and their culture supernatants and epithelial cell responses to microbe-specific secreted factors.

While many studies have used high throughput sequencing technologies to identify and characterize microbial communities present in the cervicovaginal space ^38–40^, few have leveraged whole genome transcriptomic sequencing to investigate the genes and functional pathways altered by host-microbe interactions between different vaginal bacteria and their supernatants on all three cervicovaginal epithelial cells types. The RNA-seq results from this study reveal host epithelial cell-specific genes and functional pathways that are modulated by *G. vaginalis* and L. crispatus. Whole-genome transcriptomic evaluations of ectocervical, endocervical and vaginal cells exposed to *G. vaginalis* or *L. crispatus* (or their culture supernatants) revealed unexpected findings in that *L. crispatus* supernatant exposure modulated a high number of genes while live *L. crispatus* modulated very few genes. As studies have shown that *L. crispatus*-dominated microbiota protect the cervicovaginal epithelium from viral and bacterial infection ^39,41,42^, it has been hypothesized that *L. crispatus* secreted factors contribute to this protection. For example, a previous study identified that culture supernatant from *Lactobacillus* spp., including *L. crispatus*, is protective against *Chlamydia trachomatis* infection due to the protective effects of D(−) lactic acid and a reduction in vaginal epithelial cell proliferation ^39^. Perhaps not as surprisingly, RNA sequencing revealed that live *G. vaginalis* and its culture supernatant both altered the expression of a high number of genes, with live *G. vaginalis* modulating more than its culture supernatant. The difference in gene expression profiles induced by live bacteria or their culture supernatants by these two common bacterial species demonstrates the complexity of the host-microbial interactions in the cervicovaginal space. The induction of diverse and specific molecular pathways by vaginal bacteria serve to ascribe mechanisms by which these microbes promote reproductive health or disease.

Unique transcriptomic signatures were observed for each type of cervicovaginal epithelial cell studied. After *G. vaginalis* exposure, distinct sets of genes were upregulated in each epithelial cell type indicating that most altered genes appear to be cell type specific; however, many of these genes target similar innate immune pathways providing evidence of the redundancy in immune regulation. The innate immune pathways most predominantly implicated in the cervical and vaginal epithelial cell response to *G. vaginalis* included the upregulation of NFkB signaling, an increase in anti-microbial peptides (AMPs) and activation of the NLRP3 inflammasome. The finding that *G. vaginalis* initiates an NFkB-mediated inflammatory response in cervicovaginal epithelial cells is not unexpected as previous studies by our laboratory, and many others, have shown significant increases in cervical and vaginal epithelial cell cytokine levels after *G. vaginalis* exposure ^23,43,44^. Interestingly, a recent study investigated the vaginal epithelial proteome after exposure to *G. vaginalis* culture supernatants and identified an activation of the mTOR signaling pathway ^45^. As mTOR is known to regulate many immune functions including the activation of NFkB ^46^, it is possible that mTOR may play a role in *G. vaginalis* host-microbial functions, however, we did not detect a change in mTOR gene expression in vaginal cells after exposure to either live bacteria or culture supernatants in our RNA-seq analysis. Even though many studies have identified an activated immune response in *Lactobacillus*-deplete microbial communities, characterization of this response has been highly varied and complex putting even more emphasis on the need to clearly identify specific immune factors/pathways activated by cell and microbe specific interactions.

As a critical part of the innate immune response, AMPs, also known as host defense peptides, act to destroy invading/foreign pathogens using a variety of biological processes. The four AMPs measured in this study use overlapping but distinct mechanisms of host defense including the regulation of immune cell migration (CCL20) ^47–49^, acting as a chemoattractant for neutrophils (S100A8) ^50,51^, barrier protection against neutrophil elastase (SLPI), or sequestering iron to limit bacterial nutrients and prevent growth (LCN2). *G. vaginalis*-mediated increases in CCL20 and S100A8 were most consistently observed in all three cervicovaginal cells indicating that these AMPs are likely to play an essential role in the host response. Additionally, immune cell recruitment by *G. vaginalis* through the release of CCL20 and S100A8 is a potential plausible mechanism as to how *G. vaginalis* colonization results in increased risk of HIV acquisition. Overall, these data provide evidence that the host uses multiple different mechanisms, controlled by individual AMPs, to protect against pathogenic microbes and speaks to the complexity of the innate immune response and the role of unique AMPs in this response.

Interestingly, RNA-seq identified a significant upregulation of NOD-like receptor (NLR) family, pyrin domain-containing protein 3 (NLRP3) inflammasome-associated genes in cervicovaginal cells after *G. vaginalis* exposure. Inflammasomes are a group of proteins that act as intracellular pathogen recognition receptors (PRRs) that respond to pathogen associated molecular patterns (PAMPs) from foreign microbes. Inflammasomes are activated in a multitude of inflammatory conditions, but very little is known about microbe-mediated inflammasome activation in the cervicovaginal space. Inflammasomes have been shown to be activated in immune cells after bacterial challenge with cervicovaginal microbes including *Neisseria gonorrhoeae* and *G. vaginalis* ^52,53^. Specifically, *G. vaginalis* has been shown to activate the NLRP3 inflammasome in macrophages and THP-1 monocytes ^53,54^. In this study, we confirmed that *G. vaginalis* increases caspase-1 and IL-1β levels through NLRP3 in THP-1 monocytes, an effect that is not seen with *L. crispatus*.

Noting this prior work, this is the first study to demonstrate that *G. vaginalis* activates a canonical inflammasome pathway, not just in immune cells, but also in cervicovaginal epithelial cells. *G. vaginalis* activation of the inflammasome pathway seems to be mediated, at least in part, through NLRP3, as the NLRP3 specific inhibitor, glybenclamide, was able to reduce the *G. vaginalis*-mediated increases in caspase-1 and IL-1β. While prior reports demonstrate that NLRP3 activation is essential for the induction of the inflammasome by *G. vaginalis* for immune cells, we find that the induction of the inflammasome in cervicovaginal epithelial cells is more complex ^53,55^. RNA-seq showed significant increases in expression in several genes associated with the inflammasome pathway. As such, increased NLRP1 expression in cervicovaginal cells after exposure to *G. vaginalis* suggests that NLRP1 may also be contributing to the inflammasome-mediated immune activation. Future studies would be needed to further investigate the role of NLRP1, and possibly other *G. vaginalis*-mediated inflammasome components (Supplemental Table 7), in the regulation of host-microbial interactions with *G. vaginalis*. The finding that *G. vaginalis* can activate the inflammasome in both cervicovaginal epithelial cells and monocytes suggests that inflammasome activation may be an important and common contributor to the inflammatory response in a *G. vaginalis*-dominated cervicovaginal space.

While cell death (pyroptosis) is an integral part of inflammasome activation, *G. vaginalis* also secrets a cholesterol-dependent cytolysin, vaginolysin, that creates pores in the epithelial cell membrane causing cell death ^56^. Therefore, it is difficult to discern if the cell death observed after *G. vaginalis* exposure is due to inflammasome activation directly, an indirect result of vaginolysin or a combination of both. While our results show that cell death in cervicovaginal cells is mediated by caspase-1 activation, in agreement with studies that show caspase-1 is required for Gasdermin cleavage and activation (necessary for NLRP3-induced pyroptosis) ^57^, we did not see a reduction in cell death with the NLRP3 inhibitor in either cervicovaginal cells or monocytes. Further elucidation of the mechanisms leading to cell death would be important for therapeutic strategies to limit *G. vaginalis*-mediated epithelial dysfunction.

Demonstrating the capabilities of different vaginal bacteria species to induce unique molecular profiles, in contrast to the induction of innate inflammatory pathways by *G. vaginalis*, cervicovaginal cells exposed to *L. crispatus* culture supernatant predominantly showed alterations in RNA polymerase, histone, and transcription-related genes. These findings suggest an important role for *L. crispatus* culture supernatant in inducing epigenomic modifications of host epithelial cells. An abundance of *Lactobacillus* spp. (including *L. crispatus, L. jensenii* and *L. gasseri*) in the cervicovaginal space have been shown to be protective against microbial pathogens, vaginal infection, and cervical cancer ^58^. While the biological mechanisms contributing to *Lactobacillus* protection remain largely unknown, studies have shown that *Lactobacillus* spp. produce lactic acid ^59,60^, hydrogen peroxide ^61^ and bacteriocins ^41^ that act by creating an environment that inhibits the growth of pathogenic microbes. As evidence of a direct impact of *L. crispatus* on epithelial function, *L. crispatus* has been demonstrated to inhibit cell proliferation and migration ^62^.

Unique to this study, we find that *L. crispatus* supernatant caused substantial modulation of genes globally regulating the transcriptome and epigenome. These changes corresponded to a substantial unexpected reorganization of the epigenome as shown by ATAC-seq. *L. crispatus* largely reduced the number of open chromatin regions in ectocervical cells. An intriguing hypothesis is that *L. crispatus* may be important for increasing the resilience of cells to infection (ex. Chlamydia, HIV, HPV) by modulating the epigenetic susceptibility of the cells to a disease or pathogen ^63,64^. Providing evidence for the ability of *L. crispatus* to alter the epigenome to protect from disease, a previous study has shown that supernatant from *Lactobacillus* spp. can decrease *C. trachomatis* infection by inhibiting cell proliferation through epigenetic mechanisms. Specifically, *L. crispatus* culture supernatants decreased histone deacetylase 4 (HDAC4) and increased histone acetylase EP300 through mechanisms thought to involve D(−) lactic acid in vaginal cells ^39^. Based on this report, we can hypothesize that *L. crispatus* culture supernatants induce these epigenomic effects due to the presence of lactic acid isomers. However, it is also plausible that other metabolites and/or proteins within the *L. crispatus* culture supernatant are responsible for inducing these epigenomic changes. Importantly, understanding the ability of *L. crispatus* supernatants to modify cervicovaginal epithelial function may provide new therapeutic strategies to optimize reproductive health.

Intriguingly, in this study the regions of differential chromatin accessibility did not overlap with regions previously described in published putative enhancers. This discrepancy could be due to multiple reasons. First, we do not have ENCODE or ATAC profiles of healthy primary cervical tissue for comparison. In addition, the profiled cervical cancer specimens were derived from only four specimens and likely have different biological profiles than the ectocervical lines used here. Therefore, it is possible that these differentially regulated regions are cell type-specific enhancers in ectocervical cells. Second, it is notable that there is a substantial increase in the proportion of intronic sites among the ectocervical differentially accessible sites compared to all the ectocervical peaks (48.2% vs 26.7% respectively), suggesting a potential role for Lactobacillus in regulating isoform transcriptions through chromatin modulation. However, RNA-seq data in this study is limited in its ability to directly answer this question; a repeat of this study with long read RNA-seq would be more suitable^65^. Lastly, there is a growing literature about the roles of lactate as a precursor to acetyl-CoA needed for histone acetylation as well as a direct modifier of histones through histone lactylation^66–69^. Histone lactylation is generally described to lead to increased expression, putatively associated with more open chromatin. Yet, the role of lactate in modifying histones and other epigenetic regulators is a new area of research and further work would be needed to elucidate its role in decreasing chromatin accessibility in specific contexts. Nonetheless, while we cannot fully identify how these differentially regulated sites may be contributing to gene regulation, it is notable that disease gene enrichment analysis of transcription factors associated with the motifs at these sites demonstrated multiple pathologies related to women’s health, including fertility and endometriosis, posing an intriguing avenue for better understanding the molecular underpinnings of these common but poorly understood disorders.

A limitation of this study is that it relies on single strains of common vaginal bacteria. While studies investigating broader microbial communities are needed to more accurately mimic the cervicovaginal microbiota present *in vivo*, by focusing on single microbes of interest we were able to identify specific functional pathways that may drive adverse outcomes. An in-depth understanding of single bacteria lays the foundation for these future studies. In addition to *G. vaginalis*, studies investigating other high-risk anaerobic bacteria for adverse reproductive outcomes ^7^, including *Sneathia* spp, *Mobiluncus* spp. and *Prevotella* spp. would be necessary to elucidate the combinatorial effects of anaerobes more broadly on cervicovaginal function. Now that specific biological functions of both *G. vaginalis* and *L. crispatus* have been identified, these pathways can be used to develop mechanistic hypotheses to evaluate more complex microbiota or clinical populations. For the analysis of ATAC data, publicly available databases, such as the ENCODE database, do not contain comparable ATAC data in cervical tissue or vaginal tissue for comparison of normal/control human tissue^32^. Developing these resources should be a priority as they will be critical for further understanding of the host-microbial interactions in the reproductive tract and their role in diverse reproductive outcomes.

Overall, the results of this study identify novel transcriptomic and epigenomic pathways altered by microbes within the cervicovaginal space that are most commonly associated with reproductive health and disease. Utilizing whole genome RNA-sequencing, we identified microbe-specific functional gene pathways including activation of the innate immune response by *G. vaginalis* and increased RNA transcription and histone modifications by *L. crispatus* that are regulated by host-microbe interactions within the cervicovaginal space. These results suggest that complex interactions between host cells and live bacteria or the factors they secrete have distinct and specific functions in modifying host epithelial cell responses. Novel to this study, we identified two key areas for potential therapeutic targets: 1) activation of the inflammasome as part of the innate immune response to *G. vaginalis* and 2) epigenetic regulation by *L. crispatus* culture supernatants. Targeting specific *G. vaginalis*-mediated innate immune pathways may serve to modulate the inflammatory response associated with *G. vaginalis*, and thus, could impact STIs, BV and preterm birth. Likewise, leveraging the potential of *L. crispatus* to alter the epigenetic landscape may provide new opportunities to optimize the cervicovaginal epithelial barrier and prevent pathogenic (e.g. chlamydia, HPV) microbes from harming and infecting the epithelial barrier. Continued elucidation of host-microbial interactions in the female reproductive tract will undoubtedly serve to optimize reproductive health.

## Materials and Methods

### Cell Culture

Ectocervical (Ect/E6E7, AATC# CRL-2614) (Ecto), endocervical (End1/E6E7, AATC# CRL-2615) (Endo) and vaginal (VK2/E6E7, ATCC# CRL-2616) (VK2) human epithelial cell lines (American Type Culture Collection, Manassas, VA) were cultured in Keratinocyte-Serum Free Media (K-SFM) supplemented with 0.1 ng/mL epidermal growth factor and 50 ug/mL bovine pituitary extract (Gibco, Life Technologies), 100 U/mL penicillin, and 100 μg/mL of streptomycin at 37°C in a 5% CO_2_ humidified incubator. The monocyte cell lines, THP1-Null2 (Invivogen) and THP1-KO-NLRP3 (Invivogen), which have a biallelic KO of the N-terminal region of the NLRP3 gene, were cultured according to the manufacture’s protocol. Briefly, THP1-Null2 and THP1-KO-NLRP3 cells were grown in RPMI 1640 supplemented with 2 mM L-glutamine, 25 mM HEPES (Gibco, Life Technologies), 10% heat-inactivated fetal bovine serum (Gemini Bio), 100 μg/ml Normocin^™^, 100 U/mL penicillin, and 100 μg/mL of streptomycin. Cells were maintained in T-25 flasks at 37°C in a 5% CO_2_ humidified incubator.

### Bacterial Cultures and Preparation of Bacteria-Free Supernatants

Bacterial strains, *L. crispatus* (ATCC 33197) or *G. vaginalis* (ATCC 14018), were obtained from the American Type Culture Collection (Manassas, VA). *G. vaginalis* was grown on Tryptic Soy Agar with 5% Sheep Blood plates (Hardy Diagnostics) and *L. crispatus* was grown on De Man, Rogosa and Sharpe agar (Fisher Scientific); both strains were grown in New York City III (NYCIII) broth. Bacteria were grown at 37°C in an anaerobic glove box (Coy Labs, Grass Lake, MI).

For each experiment the following bacterial growth protocol was followed: *L. crispatus* and *G. vaginalis* glycerol stocks were streaked on agar plates, as well as, into broth tubes and grown overnight. The broth starter cultures were diluted to an optical density of 0.2 and then used to inoculate 20ml working cultures, which were grown for 20 hours prior to use in experiments. Bacterial densities of the working cultures were estimated the day of the experiment based on optical density readings at 600 nm using an Epoch2 plate reader (Biotek, Winooski, VT), and the appropriate volume was centrifuged at 13,000 × *g* for 3 min. The bacterial pellets were resuspended in the appropriate cell culture media without antibiotics and added to epithelial cells at 10^4^ −10^6^ CFUs/well. Precise bacterial densities of the working cultures were determined by plating serial dilutions of the working cultures and counting CFUs. For all experiments, reported bacterial densities are +/− 0.5 log of the noted bacterial density (CFU/well).

To obtain bacteria-free culture supernatants, the working cultures were centrifuged at 13,000 × *g* for 3 min and the supernatant was filtered through a 0.22 μm filter (Fisher Scientific) to remove any remaining live bacteria. Bacteria-free culture supernatants were diluted to 1% v/v in the appropriate cell culture media without antibiotics.

### In vitro Epithelial and Immune Cell - Bacteria Interactions

Ectocervical, endocervical and vaginal cells were plated at 1.5 × 10^5^ cells/well in twenty-four well plates containing K-SFM without antibiotics. THP1-Null2 or THP1-KO-NLRP3 cells were plated at 1.5 × 10^5^ cells/well in twenty-four well plates containing RPMI 1640 media without antibiotics. The next day, the cells were exposed to either live *L. crispatus* or *G. vaginalis* (1×10^5 −^ 1×10^7^ CFU/well) or 1% (v/v) bacteria-free supernatants (generated from a 1×10^7^ CFU/mL culture) for 24 hr. For cells treated with bacteria-free supernatants from *L. crispatus*, K-SFM media was supplemented with 50mM HEPES and sodium bicarbonate (3000 mg/L total concentration) to bring the pH of the media up to a physiological level (7.2). In additional experiments, ectocervical (n = 6/treatment), endocervical (n = 6/treatment), vaginal cells (n = 6/treatment) or THP1-Null2 (n = 6/treatment) cells were pre-treated with Glybenclamide (100 μg/ml, Invivogen), a specific NLRP3 inhibitor, for 30 mins prior to live bacteria exposure or Ac-YVAD-cmk (25 μM, Sigma-Aldrich), an irreversible caspase-1 inhibitor, for 1 hour prior to live bacteria exposure. For inflammasome experiments, LPS (10 ng/ml) exposure for 24 hr was used as a positive control of inflammasome activation. For all supernatant experiments, cells were also exposed to NYCIII bacterial growth media alone as a negative control to determine any baseline effects of the growth media on the outcomes of interest. At the end of each experiment, cell culture media was collected for cell death, ELISA assays and/or the cells were collected in Trizol (Invitrogen, Thermo-Fisher Scientific) for RNA extraction.

### RNA Sequencing and Analysis

RNA was extracted from ectocervical, endocervical and vaginal cells after exposure to bacteria or culture supernatants from *L. crispatus* and *G. vaginalis* (n = 3/treatment group) collected in Trizol using the Qiagen-RNeasy Plus Mini kit by the Penn Next-Generation Sequencing Core. The resulting RNA had RIN values > 9. Illumina sequencing libraries were prepared using the Illumina TruSeq mRNA stranded library prep kit according to the manufacturer recommendations. The resulting libraries had an average molarity of 69 nM +/1 27 nM. Libraries were sequenced to a median depth of 41 million 100 bp single reads on an Illumina NovaSeq 6000. Transcript quantification from RNA-seq data was performed using Salmon and release 38 (GRCh38.p13) of the human genome ^70,71^. Several Bioconductor packages in R were used for subsequent steps ^72,73^. The output was annotated and summarized using tximeta and further annotation was completed with biomaRt ^74,75^. Principle Component Plots (PCA) were created using pcaExplorer ^76,77^. The normalizations and statistical analyses were done with DESeq2 ^78^. The full RNA-seq dataset was submitted to Gene Expression Omnibus (accession # GSE234837).

### RNA-seq Pathway Analysis

PathfindR was used for pathway enrichment analysis using Gene Ontology terms (https://github.com/egeulgen/pathfindR) ^79^. Upregulated and downregulated genes were grouped together for each comparison. The enrichment threshold was set at an FDR of 0.05, with a significant gene threshold of 0.02. A heatmap for enrichment scores for each comparison was created by first calculating and aggregating term scores for each sample included for each comparison and then averaging the scores across all compared samples. ComplexHeatmap package in R was then used to visualize the comparison of GO term analysis (rows) for all the comparisons (columns). Rows were clustered by the “complete” method with a kmeans = 10. A word cloud was used to represent the most significant recurring pathways in a cluster. Generic terms were excluded from word cloud (“pathway,” “cellular,” “regulation,” “positive,” “negative,” “cell,” “complex,” “process,” “factor,” “activity,” “protein,” “DNA,” “RNA,” “levels,” “binding,” “response,” “signaling,” “receptor”).

### Cell Death Assay

Ectocervical, endocervical, vaginal, THP1-Null2 and THP1-KO-NLRP3 cells were grown and exposed to bacteria or bacterial culture supernatants as described above. Lactate dehydrogenase (LDH) released upon cell lysis (n = 3–9 independent experiments per cell type) was quantified using the CytoTox 96 Non-radioactive cytotoxicity assay (Promega, Madison, WI), a coupled enzymatic assay that results in the conversion of a tetrazolium salt into a red formazan product. The colorimetric output was measured using a plate reader at 490 nm and absorbance values were recorded.

### ELISA

Ectocervical, endocervical, vaginal, THP1-Null2 and THP1-KO-NLRP3 cells were cultured in 24-well plates and exposed to either live bacteria or bacterial supernatants as stated above. Anti-microbial peptides, CCL20, SLPI, LCN2, S100A8/A9, or inflammasome-associated cytokines, IL-1β and caspase-1, were measured in cell culture media after 24 hr of exposure (n = 6/group). The expression of these analytes was measured by a ligand-specific commercially available ELISA kit that utilizes a quantitative sandwich enzyme immunoassay technique using reagents from R&D Systems (Minneapolis, MN).

### ATAC-seq Nuclei Extraction, Tagmentation, Purification and Library Amplification

ATAC-seq was performed on ectocervical, endocervical and vaginal cells after exposure to *L. crispatus* bacteria-free supernatants (n = 3/treatment group). ATAC-seq libraries were generated using the ATAC-seq Kit from Diagenode (Diagenode, A Hologic Company) according to manufacturer instructions. Briefly, nuclei were extracted from 50,000 cells. Tagmentation was completed by resuspending the isolated nuclei in transposase reaction mix and the samples were purified using the kit’s provided columns. Following purification, library fragments were amplified by PCR according to the manufacturer recommendations. Unique Dual Indexes Primer Pairs were incorporated for multiplexed sequencing. To reduce amplification bias, after the first 5 cycles of the PCR reaction, qPCR was used to determine how many additional cycles were needed to produce enough library to meet the required amount for sequencing. For this, an aliquot of the PCR reaction was added to Sybr Green and amplified for 20 cycles. Libraries were amplified for a total of 11–13 cycles (with one library requiring 17 cycles for amplification). Final libraries were purified using bead purification (Beckman Coulter), then assessed for size distribution and concentration using a BioAnalyzer High Sensitivity DNA Kit (Agilent Technologies). The resulting libraries were pooled. The pool was diluted to 2 nM, denatured, and then 13 libraries were loaded onto an S1–100 (2×50) flow cell on an Illumina NovaSeq 6000 (Illumina, Inc.) according to the manufacturer’s instructions. The average read number per sample was 50M+/− 20%. De-multiplexed and adapter-trimmed sequencing reads were generated using bcl2fastq. The full ATAC-seq dataset was submitted to Gene Expression Omnibus (accession # GSE233444).

### ATAC-seq Mapping and Peak Calling

ATAC-seq data analysis was adapted from a previously published approach ^26^. Each cervicovaginal cell type was analyzed separately. In brief, raw FASTQ files were processed and mapped to release 38 (GRCh38.p13) of the human genome using the PEPATAC pipeline ^80^. Reads were trimmed with *Skewer* and then aligned with *Bowtie2* using default settings ^81,82^. Duplicate reads were removed using *samblaster* ^83^.

An iterative overlap peak calling strategy on fixed-sized peaks of 501 bp was used to define a set number of peaks for each cell type for downstream differential accessibility comparison ^26^. First, for each biological replicate, MACS2 was used to call peaks with the parameters as follows: --peak-type fixed --extend 250 ^84^. Biological replicates of each treatment and then both treatments together from each cell type were merged using an iterative overlap approach previously described ^26,34^. Blacklisted regions were excluded from called peaks (accessed 4 November 2022 at https://github.com/Boyle-Lab/Blacklist) ^85^.

### ATAC-seq Peak and Differential Accessibility Analysis

Peak location was annotated with *CHIPseeker* ^86^. Counts for peaks were calculated using *Rsubread* ^87^. We determined the differential accessibility of peaks between treatments with *DESeq2* ^88^. We compared *L. crispatus* culture supernatant treated to NYCIII media controls for each cell type. A Wald test was used to determine significance. A peak was defined as statistically significant in differential accessibility if |log2foldchange| > 1 and FDR < 0.05. We utilized the R package rGREAT for the nearest gene analysis to access the Genome Regions Enrichment of Annotations Tool (GREAT) web service^29–31^. For GREAT, we used the parameters for “the two closest genes” to a differential accessible site as it is frequently not the closest genes that is differentially regulated.

Motif analysis was performed using Simple Enrichment Analysis version 5.5 as part of the MEME Suite (https://meme-suite.org/meme/tools/sea) ^89,90^. CIS-BP 2.0 motifs were used for the query ^91^. Gene-disease enrichment was performed using *disgenet2R* (https://www.disgenet.org/) ^92^. Random gene lists for comparison were generated by sampling 497 transcription factors from the CIS-BP 2.0 database.

### ATAC-seq Chromatin Accessibility Visualization

*EasSeq* was utilized to visualize the data ^93^. Biological replicates of BAM files were pooled for quantification of specific regions. Quantile normalization was used for counts per region for visualization to minimize bias from sequencing depth. Calculation of overlap performed both by any amount of overlap and the exact overlap of base pairs between all comparisons. Random regions matched of equal number comparisons to differentially accessible regions or all ectocervical open chromatin regions were generated by Regulatory Sequence Analysis Tools (random genome fragments tool; http://rsat.sb-roscoff.fr/)^94^. ENCODE datasets for all human enhancer-like sequences (ELS, defined as high DNAse-seq signal and high H3K37me3), or tissue-specific regulators were obtained from https://screen.encodeproject.org/ ^32,33^. For uterus and vaginal specimens, downloaded with “Low-DNase” were filtered out to enrich for sites that had any evidence of potential enhancer or regulator activity. However, strict enhancer-like signature criteria could not be applied because all sequencing modalities were not available for all the samples. Primary cancer cell data sets were obtained from supplemental of published ATAC profiling^34^.

### Statistical Analysis

Statistical analyses were performed for all experiments (except for RNA or ATAC sequencing, statistical analysis is described above for each) with the GraphPad Prism Software (Version 9.0, San Diego, CA). For data that were normally distributed (as assessed by Shapiro-Wilk test), one-way analysis of variance (ANOVA) was performed. If statistical significance was reached (p < 0.05), then pair-wise comparison with a Tukey post hoc test was performed for multiple comparisons. If data were not normally distributed, then the non-parametric Kruskal-Wallis test was used and pairwise comparison was done using Dunn’s multiple comparison test. Chi test for trend was utilized to compare overlaps of indicated ectocervical peaks with the number of a random set of sites matched for size and CG content.

## Figures and Tables

**Table 1:** Altered Inflammasome-related genes of interest after exposure to live L. crispatus or G. vaginalis

	Ectocervical		Endocervical		Vaginal	
Gene of Interest	LC	GV	LC	GV	LC	GV
NLRP3 associated genes						
NLRP3	0.01	1.44	0.17	1.28	0.08	1.82
NOD1	0.06	0.02	0.32	0.30	−0.05	−0.01
NOD2	−0.11	0.41	1.13	2.29	−0.42	0.17
NEK7	−0.09	0.26	−0.38	−0.39	0.02	0.07
NLRP1 associated genes						
NLRP1	0.008	0.36	0.29	1.21	0.13	0.72
Canonical Inflammasome genes						
Caspase-1	0.15	0.29	−0.17	0.79	−0.31	−0.59
IL-1B	0.16	0.96	0.31	2.06	0.15	1.37
IL-18	−0.01	0.41	−0.22	−0.46	0.16	0.10
IL-1a	0.12	1.29	0.30	2.07	0.23	1.12

Altered genes after exposure to live bacteria are shown as Log2FC from the non-treated control (NTC), gray shading denotes p≤0.01
